# Supplementary figures and images for: Cistanche promotes the adipogenesis of 3T3-L1 preadipocytes
Source: PLoS One. 2022 Mar 1;17(3):e0264772. doi: 10.1371/journal.pone.0264772 (PMC8887766; doi:10.1371/journal.pone.0264772)

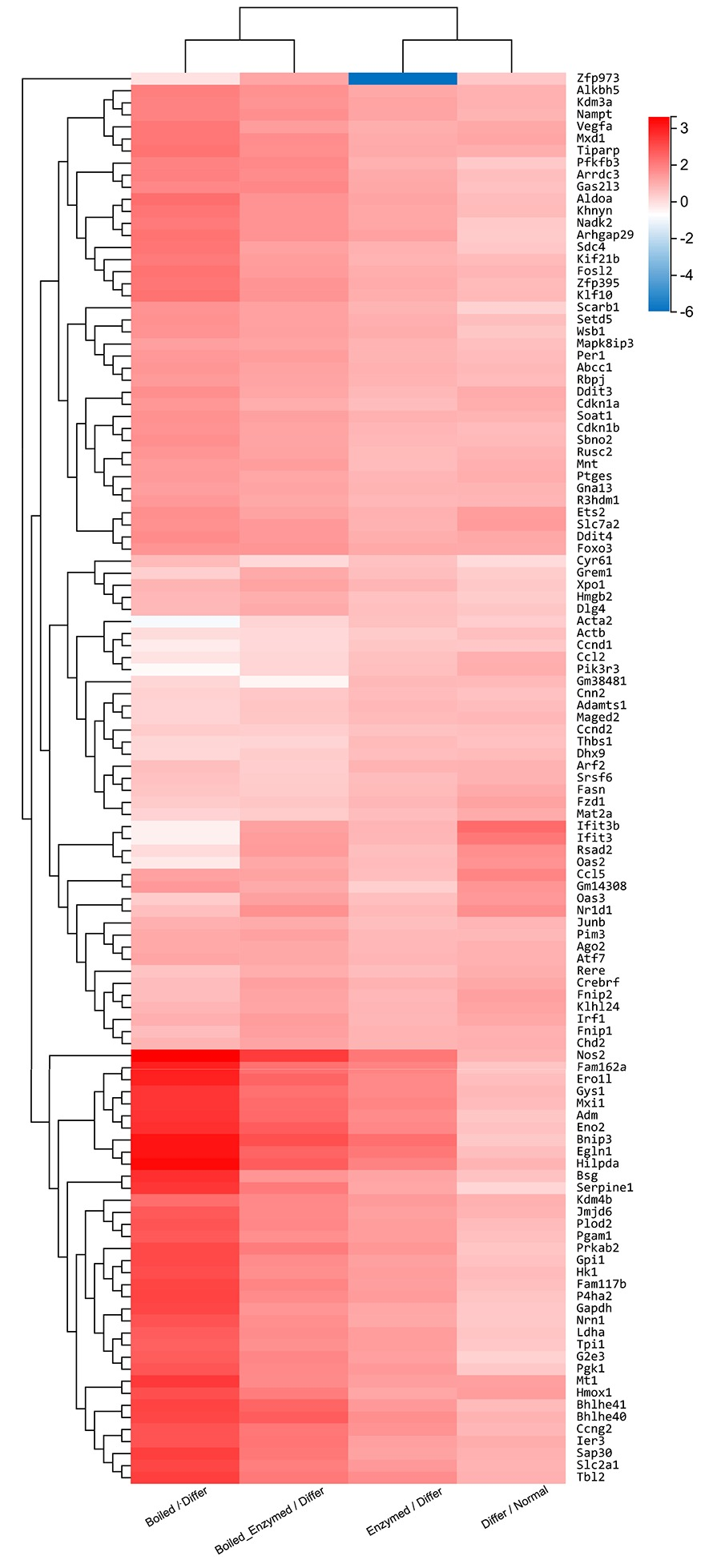

Supplement: S1 Fig — DEGs were presented in heatmap. Red indicates high expression genes, while blue indicates low expression genes. Color changing from red to blue indicate that log 10 (FPKM+1) gradually changes from big to small. Nor: Normal group. Differ: 3T3-L1 cells were treated with differentiation medium. Boiled: 3T3-L1 cells were treated with cistanche of Boiled. Enzymed: 3T3-L1 cells were treated with cistanche of Enzymed. Boiled with Enzyme: 3T3-L1 cells were treated with cistanche of Boiled with enzymed. (TIF) [file pone.0264772.s001.tif]

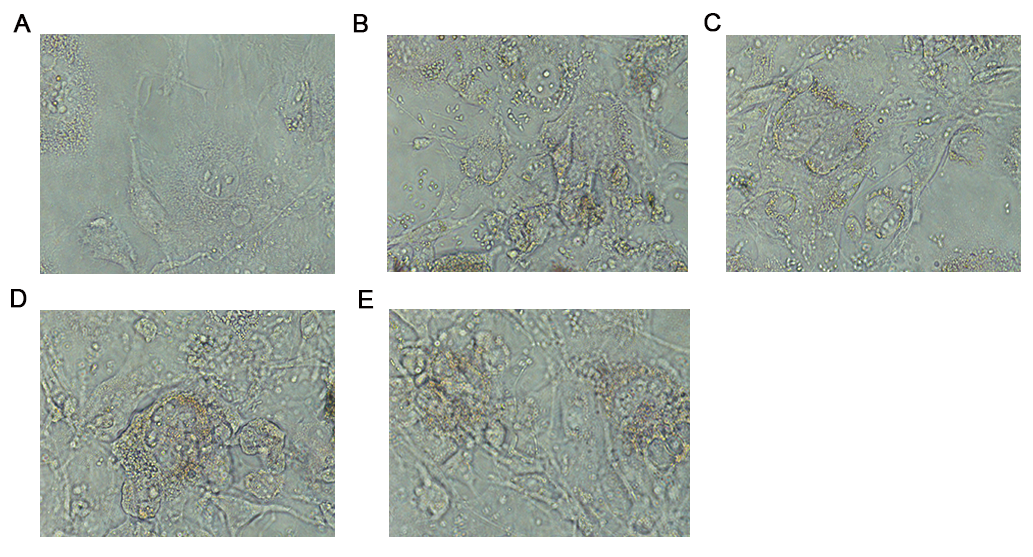

Supplement: S2 Fig — The figure of 3T3-L1 cells in pre-staining. (A) Normal group. (B) 3T3-L1 cells were treated with differentiation medium. (C) 3T3-L1 cells were treated with cistanche of Boiled. (D) 3T3-L1 cells were treated with cistanche of Enzymed. (E) 3T3-L1 cells were treated with cistanche of Boiled with Enzymed. (TIF) [file pone.0264772.s002.tif]

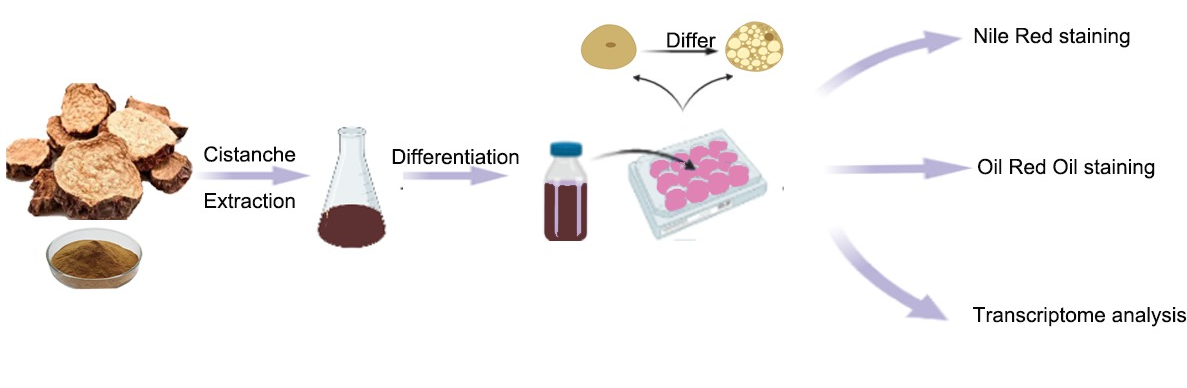

Supplement: S1 Graphical abstract — (TIF) [file pone.0264772.s003.tif]
